# Highly Efficient Removal of Uranium from an Aqueous Solution by a Novel Phosphonic Acid-Functionalized Magnetic Microsphere Adsorbent

**DOI:** 10.3390/ijms232416227

**Published:** 2022-12-19

**Authors:** Jizhou Zhao, Peng Lu, Tengteng He, Jing Huang, Shiao Zhang, Yan Liu, Yun Wang, Cheng Meng, Dingzhong Yuan

**Affiliations:** Jiangxi Province Key Laboratory of Polymer Micro/Nano Manufacturing and Devices, East China University of Technology, Nanchang 330013, China

**Keywords:** uranium elimination, magnetic polymer microsphere, phosphonic acid, radioactive water treatment

## Abstract

The development of adsorption materials which can efficiently isolate and enrich uranium is of great scientific significance to sustainable development and environmental protection. In this work, a novel phosphonic acid-functionalized magnetic microsphere adsorbent Fe_3_O_4_/P (GMA-MBA)-PO_4_ was developed by functionalized Fe_3_O_4_/P (GMA-MBA) prepared by distill-precipitation polymerization with O-phosphoethanolamine. The adsorption process was endothermic, spontaneous and kinetically followed the pseudo second-order model. The maximum uranium adsorption capacity obtained from the Langmuir model was 333.33 mg g^−1^ at 298 K. In addition, the adsorbent also had good acid resistance and superparamagnetic properties, which could be quickly separated by a magnetic field. XPS analysis showed that the adsorption of adsorbent mainly depended on the complexation of phosphonic acid group with uranium. This work offers a promising candidate for the application of magnetic adsorbents in the field of uranium separation and enrichment.

## 1. Introduction

With the intensification of the energy crisis, nuclear energy as a kind of clean energy has been widely studied [1,2,3]. The demand for uranium as an important nuclear fuel is increasing. However, uranium is characterized by radioactivity and heavy metal toxicity. If it accumulates in large quantities in the environment, it will do harm to the environment. Therefore, it is important to develop the separation technology for the efficient separation and enrichment of uranium for the exploitation of uranium resources and environmental protection [4,5,6]. At present, many methods including ion exchange, solvent extraction, chemical precipitation, membrane separation, photocatalysis and adsorption have been used to recover uranium from an aqueous solution [7,8,9,10,11,12]. Among these methods, adsorption has proved to be a promising separation technique due to its wide source of materials, low cost, high adsorption selectivity and high volumetric value [13,14,15]. The key to the adsorption method is the choice of adsorption material. At present, many materials such as carbon materials, silicon materials, polymer materials and metal–organic frames have been used in the field of uranium adsorption. However, these materials often suffer from complicated separation steps such as filtration and centrifugation, which hinder their application in the field of uranium adsorption to a great extent. Therefore, the development of a uranium adsorption medium with a high adsorption efficiency and easy separation is one of the current research key points in the field of environmental radiochemistry [16,17].

Recently, magnetic polymer nanomaterials have received much attention [18,19,20,21,22]. On the one hand, the nano size can make this material have a larger specific surface area and more surface atoms than ordinary materials, thus showing strong adsorption and performance. On the other hand, magnetic polymer nanomaterials can be separated quickly by magnetic force, which solves the problem of the separation difficulty of traditional adsorption materials [23,24,25,26,27,28,29,30,31]. In addition, the surface of the material can also be rich in organic functional groups by means of copolymerization or post-modification, which can improve the adsorption capacity for uranium. Therefore, magnetic polymer nanomaterials have obvious advantages in the separation and enrichment of uranium, which cannot be replaced by conventional materials in many aspects. Based on these advantages, the development of magnetic polymer nanomaterials has important scientific significance and practical value for the healthy and rapid development of the nuclear industry.

Up to now, as an efficient ligand for uranium, the phosphonic acid group has been widely used for the separation and enrichment of uranium due to its strong complexing ability with uranyl ion. For example, in our preliminary work, many phosphonic acid-functionalized adsorbents were prepared by solvothermal polymerization, distillation precipitation polymerization and other methods, and all of them had good adsorption properties for uranium [1,16]. In addition, Broda et al. [32] reported a nanocomposite hydroxyapatite/white clay with excellent adsorption properties for uranium. Therefore, considering these factors, this work hoped to develop a phosphonic acid-functionalized magnetic polymer microsphere adsorbent for the efficient separation and enrichment of uranium from aqueous solutions [33,34].

In this work, a novel phosphonic acid-functionalized magnetic polymer microsphere adsorbent Fe_3_O_4_/P (GMA-MBA)-PO_4_ was developed by functionalizing Fe_3_O_4_/P (GMA-MBA) prepared by distill-precipitation polymerization with O-phosphoethanolamine. Surprisingly, the Fe_3_O_4_/P (GMA-MBA)-PO_4_ showed a good adsorption capacity, with the theoretical maximum adsorption capacity of uranium reaching 333.33 mg g^−1^ at pH 4.5. In addition, the adsorbent also had good structural stability and superparamagnetic character, resulting in the quick separation and recovery by magnetic force of the adsorbent. XPS analysis showed that the adsorption of the adsorbent mainly depended on the complexation of the phosphonic acid group with uranium. This work offers a promising candidate for the application of magnetic adsorbents in the field of uranium separation and enrichment.

## 2. Results and Discussion

### 2.1. Structural Analyses

**Scheme 1:**
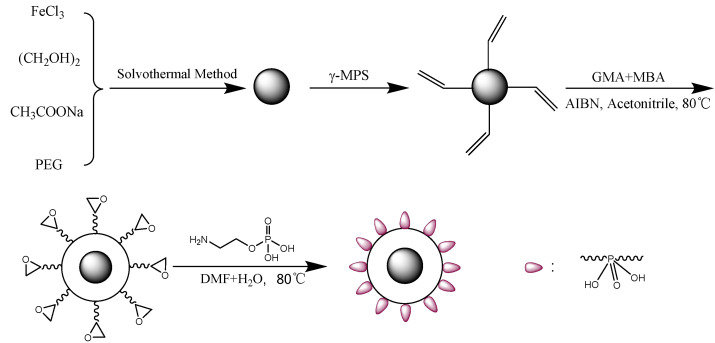
Proposed synthesis of the magnetic adsorbent Fe_3_O_4_/P (GMA-MBA)-PO_4_.

To verify that Fe_3_O_4_/P (GMA-MBA)-PO_4_ was successfully synthesized by Scheme 1, the physical and chemical properties of the adsorbent were characterized by TEM, FT-IR, XRD, VSM and TGA, respectively.

**Figure 1:**
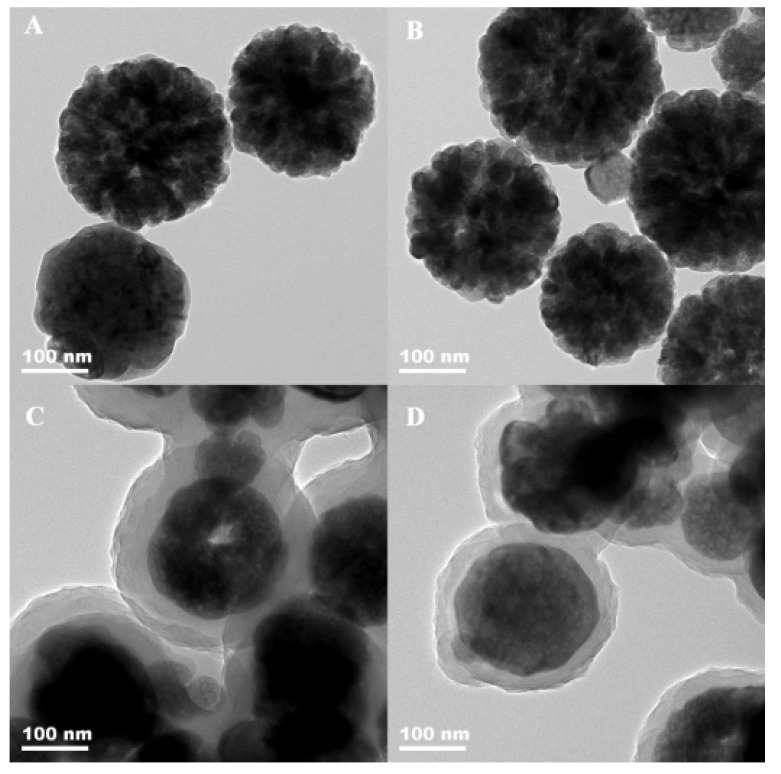
The TEM images for (**A**) Fe_3_O_4_, (**B**) activated Fe_3_O_4_, (**C**) Fe_3_O_4_/P (GMA-MBA) and (**D**) magnetic adsorbent Fe_3_O_4_/P (GMA-MBA)-PO_4._

The morphology of Fe_3_O_4_, activated Fe_3_O_4_, Fe_3_O_4_/P(GMA-MBA) and the magnetic adsorbent Fe_3_O_4_/P (GMA-MBA)-PO_4_ were investigated using TEM. As shown in Figure 1A, the prepared Fe_3_O_4_ had a uniform particle size with an average diameter of about 200 nm. The magnetic sphere surface had a fluffy structure [35]. The mean particle size and morphology of Fe_3_O_4_ did not change significantly after the modification of γ-MPS (Figure 1B), suggesting that the activation process did not have much effect on the morphology of Fe_3_O_4_. Figure 1C shows that there was a thick polymer shell layer around the Fe_3_O_4_, and the thickness of the polymer shell was around 35 nm, indicating that the copolymerization of GMA and MBA had occurred smoothly on the surface of Fe_3_O_4_ to obtain the matrix material Fe_3_O_4_/P (GMA-MBA). Figure 1D lists the TEM image of Fe_3_O_4_/P (GMA-MBA)-PO_4_. From Figure 1D, the magnetic adsorbent still maintained the core–shell structure, indicating the matrix material Fe_3_O_4_/P (GMA-MBA) had an excellent medium resistance.

**Figure 2:**
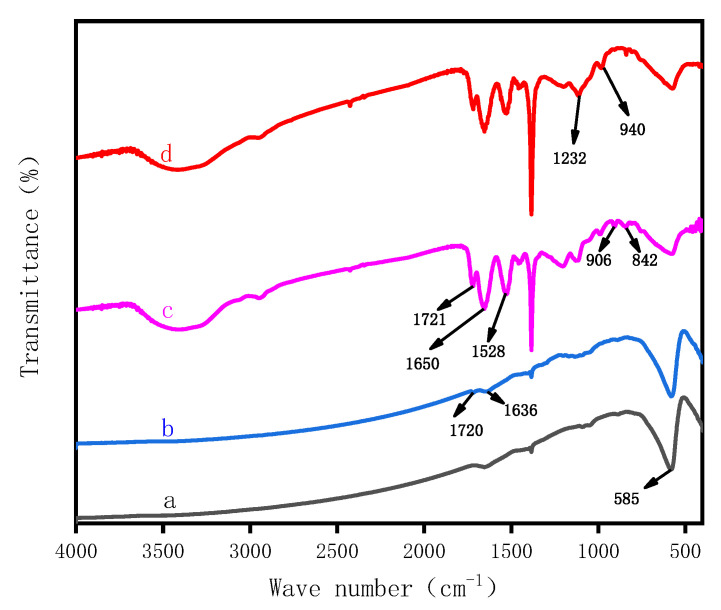
Infrared spectra of (**a**) Fe_3_O_4_; (**b**) activated Fe_3_O_4_; (**c**) Fe_3_O_4_/P (GMA-MBA) and (**d**) Fe_3_O_4_/P (GMA-MBA)-PO_4._

Figure 2 shows the FTIR spectra of Fe_3_O_4_, Fe_3_O_4_ activated by KH570, Fe_3_O_4_/P(GMA-MBA) and Fe_3_O_4_/P (GMA-MBA)-PO_4_. As shown in Figure 2a, the characteristic peaks located around 585 cm^−1^ belonged to the characteristic absorption of Fe-O in Fe_3_O_4_. Compared to Fe_3_O_4_, a series of new peaks ascribed to γ-MPS can be observed in Figure 2b. For example, tow new peaks located at approximately, 1636 cm^−1^ and 1720 cm^−1^ were attributed to the -C=C and -C=O groups of γ-MPS, respectively, indicating that Fe_3_O_4_ had been functionalized by KH570 successfully [36]. In the spectrum of Fe_3_O_4_/P (GMA-MBA) (Figure 2c), many new characteristic absorption bands ascribed to P (GMA-MBA) could be observed. For instance, the two obvious peaks located at 1528 cm^−1^ and 1650 cm^−1^ belonged to the stretching vibration of N-H and C=O from MBA. In addition, the three obvious peaks located at 842 cm^−1^, 906 cm^−1^ and 1721 cm^−1^ belonged to the stretching vibration of epoxy group and C=O from GMA. Thus, all these results indicated that the co-polymerization of GMA and MBA had occurred on the surface of activated Fe_3_O_4_. Figure 2d shows the infrared spectrum of Fe_3_O_4_/P(GMA-MBA)-PO_4_. The two obvious peaks located at 940 cm^−1^ and 1232 cm^−1^ belonged to the stretching vibration of P-OH and P=O [37]. Besides, the epoxy group characteristic absorption peak (842 cm^−1^ and 906 cm^−1^) appearing in Figure 2c almost disappeared. Thus, all these results suggest that Fe_3_O_4_/P(GMA-MBA) was successfully modified with O-phosphoethanolamine.

**Figure 3:**
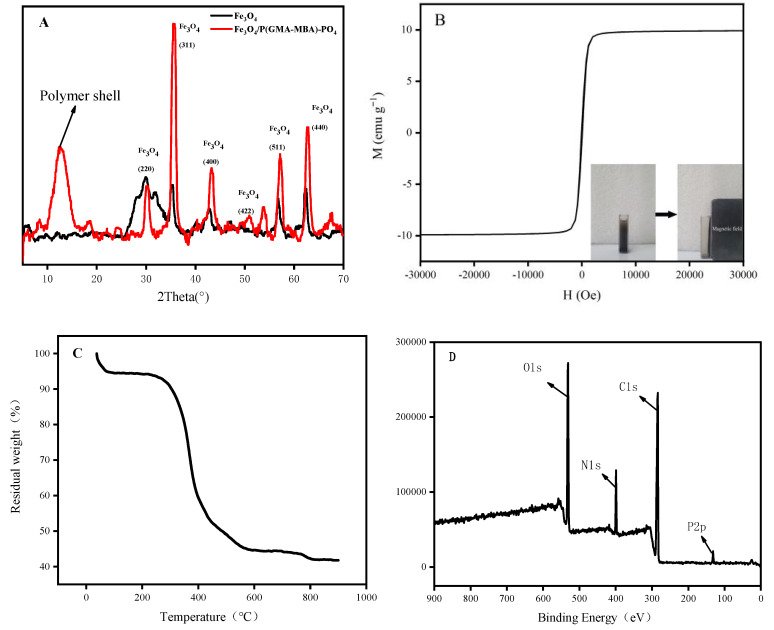
(**A**) XRD pattern of Fe_3_O_4_, and Fe_3_O_4_/P(GMA-MBA)-PO_4_; (**B**) VSM curve; (**C**) TGA curve and (**D**) XPS spectrum of Fe_3_O_4_/P(GMA-MBA)-PO_4._

The X-ray powder diffraction (XRD) pattern of Fe_3_O_4_ and Fe_3_O_4_/P(GMA-MBA)-PO_4_ was shown in Figure 3A. The XRD pattern of Fe_3_O_4_/P(GMA-MBA)-PO_4_ showed many characteristic peaks of Fe_3_O_4_. Moreover, a peak of dispersion at a 2θ of about 12.5° representing amorphous P(GMA-MBA) was also seen in addition to the characteristic peaks of Fe_3_O_4_. The VSM pattern of Fe_3_O_4_/P (GMA-MBA)-PO_4_ is shown in Figure 3B. As shown in Figure 3B, the hysteresis loop passed through the origin with a coercivity force of zero, confirming the magnetic polymer microsphere adsorbent was superparamagnetic with a specific saturation magnetization of about 10.0 emu g^−1^. Thus, Fe_3_O_4_/P (GMA-MBA)-PO_4_ could be quickly separated and recovered from the aqueous solution by applying an external magnetic field within 20 s (Figure 3B inset). The thermal stability of the material was tested, and the results are shown in Figure 3C. From Figure 3C, the lost weight of the sample was mainly caused by the volatilization of the small molecule including water, solvent and monomers remaining on the surface of Fe_3_O_4_/P (GMA-MBA)-PO_4_ within 50–293 °C. However, when the temperature was above 293 °C, the mass of the magnetic adsorbent dropped sharply, which might have been due to the oxidative degradation of the polymer chain. Thus, according to Figure 3C, the prepared magnetic adsorbent had a high thermal stability. As shown in Figure 3D, the elemental composition of Fe_3_O_4_/P (GMA-MBA)-PO_4_ was also analyzed by XPS, and the magnetic adsorbent contained C, N, O and P. Combined with the results of infrared spectroscopy analysis, it is clear that the phosphonic acid group was successfully introduced to the surface of the magnetic sphere. Based on these results, the phosphonic acid functionalized core–shell magnetic sorbent Fe_3_O_4_/P(GMA-MBA)-PO_4_ was successfully prepared.

### 2.2. Effect of pH

**Figure 4:**
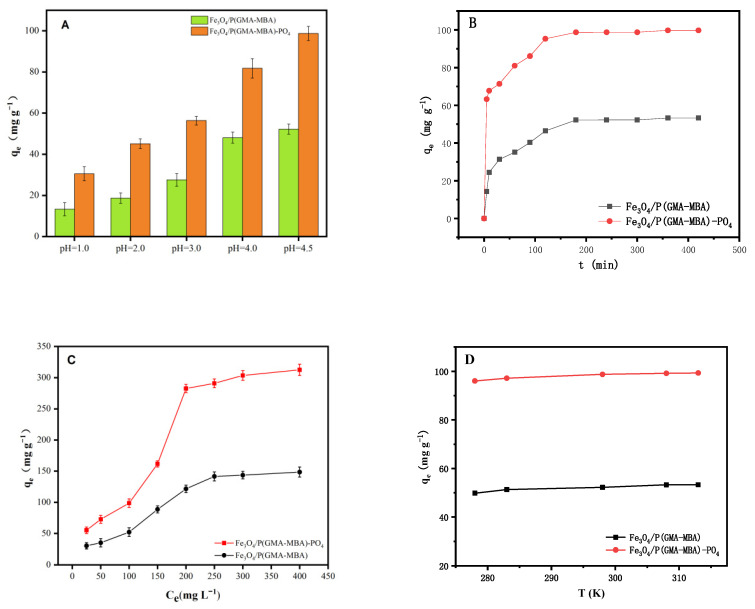
(**A**) Adsorption performance of Fe_3_O_4_/P (GMA-MBA) and Fe_3_O_4_/P (GMA-MBA)-PO_4_ on uranium under different pH values; (**B**) influence of contact time on uranium adsorption capacity (C_0_ = 100 mg L^−1^, T = 298 K, pH = 4.5, m/V = 0.4 g L^−1^); (**C**) effect of uranium concentration on adsorption capacity of Fe_3_O_4_/P (GMA-MBA) and Fe_3_O_4_/P (GMA-MBA)-PO_4_ (T = 180 min, pH = 4.5, T = 298 K, m/V = 0.4 g L^−1^); (**D**) thermodynamic image of U (VI) adsorbed by Fe_3_O_4_/P (GMA-MBA) and Fe_3_O_4_/P (GMA-MBA)-PO_4_ (C_0_ = 100 mg L^−1^, pH = 4.5, m/V = 0.4 g L^−1^).

In general, the adsorption capacity of uranium is often significantly affected by pH, due to which the speciation of uranium and the surface charge of the adsorbent are affected to a large extent. Hence, the uranium adsorption capacity on Fe_3_O_4_/P (GMA-MBA) and Fe_3_O_4_/P (GMA-MBA)-PO_4_ was investigated with an initial pH ranging from 1.0 to 4.5, and the experimental results are shown in Figure 4A. From Figure 4A, the uranium adsorption capacity of the two magnetic adsorbents increased with the value of pH. In aqueous solutions of pH 4.5, the adsorption capacity of Fe_3_O_4_/P (GMA-MBA) and Fe_3_O_4_/P (GMA-MBA)-PO_4_ could reach up to 52.21 and 98.73 mg g^−1^, respectively. From Figure 4A, the uranium adsorption capacity of Fe_3_O_4_/P (GMA-MBA)-PO_4_ was much higher than that of Fe_3_O_4_/P (GMA-MBA). This result confirms that the prepared magnetic polymer microsphere adsorbent had the ability to efficiently separate the enriched uranyl ions in the aqueous solution, which was mainly due to the complexation of phosphoric acid functional groups on the surface of the adsorbent with uranyl ions.

As shown in Figure 4A, under highly acidic conditions (at pH < 2), the U (VI) adsorption capacity of Fe_3_O_4_/P(GMA-MBA)-PO_4_ was very low. This was mainly because the material surface group was protonated when the solution pH value was very low, and the adsorbent surface was positively charged. Since uranium (VI) mainly exists in the form of UO_2_^2+^ when the pH value was less than 4.5 (Figure S1), electrostatic repulsion between the adsorbed material and UO_2_^2+^ reduced the adsorption performance of uranium. When the acidity of the system decreased, the protonation degree of the adsorbent surface reduced. In such a case, the electrostatic repulsion between the adsorption material and UO_2_^2+^ was constantly reduced, and the complexation ability of the functional group of the adsorbent surface with uranyl ion was enhanced, resulting in the increase in the uranium adsorption capacity of the adsorbent. In addition, when the value of pH was greater than 4.5, uranium became unstable and easy to precipitate. Therefore, pH of 4.5 was applied as the best operation condition for further experiments.

### 2.3. Sorption Static Kinetics, Isotherms and Thermodynamic Analysis

To acquire sorption kinetics, isotherms and thermodynamic data, the effect of time, uranium concentration and temperature on the adsorption capacity of the adsorbent for uranium was evaluated, and the results are listed in Figure 4B–D. 

The uranium adsorption capacity of Fe_3_O_4_/P(GMA-MBA) and Fe_3_O_4_/P(GMA-MBA)-PO_4_ at different adsorption times is shown in Figure 4B. From Figure 4B, the adsorption of uranium at pH 4.5 could be roughly divided into three stages. In the first 120 min, the adsorption rate was very fast and the adsorption capacity increased rapidly. At 120–180 min, the adsorption rate was reduced. The adsorption was gradually balanced after 180 min. Since the Fe_3_O_4_/P(GMA-MBA)-PO_4_ was rich in the phosphate acid group, the saturation adsorption capacity of Fe_3_O_4_/P(GMA-MBA)-PO_4_ was greater than that of Fe_3_O_4_/P(GMA-MBA).

Table S1 lists the kinetic parameters obtained by pseudo-first-order, pseudo-second-order and intra-particle diffusion models [38,39,40]. According to Table S1 and Figures S2–S4, the adsorption process was better correlated with the pseudo-second-order kinetic models. Calculated from the linear equation of the pseudo-second-order, the theoretical adsorption volume q_e_ values of Fe_3_O_4_/P(GMA-MBA) and Fe_3_O_4_/P(GMA-MBA)-PO_4_ at pH 4.5 were 52.91 mg g^−1^ and 101.21 mg g^−1^, very close to the actual experimental results (52.21 mg g^−1^ and 98.73 mg g^−1^). This showed that the pseudo-second-order kinetic model was better suited for describing the adsorption process of uranium by Fe_3_O_4_/P(GMA-MBA)-PO_4_, since the pseudo-second-order model is based on the assumption that the adsorption rate is controlled by chemical adsorption. Thus, the adsorption mechanism of uranium by Fe_3_O_4_/P (GMA-MBA)-PO_4_ and Fe_3_O_4_/P(GMA-MBA) in pH 4.5 solution was mainly dominated by chemical adsorption. 

The effect of the initial concentration of uranyl ion on the uranium adsorption capacity of Fe_3_O_4_/P(GMA-MBA) and Fe_3_O_4_/P(GMA-MBA)-PO_4_ in aqueous solutions at pH 4.5 was investigated, and the results are shown in Figure 4C. From Figure 4C, the adsorption capacity of the two magnetic adsorbents increased as the concentration of the uranium solution increased from 25 mg L^−^^1^ to 200 mg L^−^^1^. When the concentration of the solution reached above 250 mg L^−^^1^, the adsorption capacity of the two magnetic adsorbents increased. When the concentration was 300 mg L^−^^1^, the equilibrium adsorption capacity of Fe_3_O_4_/P (GMA-MBA) reached 148.59 mg g^−^^1^. When the adsorption capacity of Fe_3_O_4_/P (GMA-MBA)-PO_4_ increased to 303.59 mg g^−^^1^, the adsorption capacity remained unchanged with the continued increase in concentration. The main reason for this was that when the concentration of uranium ions in solution increased, the adsorption sites on the surface of the adsorbent were occupied by uranyl ions. When the concentration increased to a certain extent, the adsorption sites were occupied completely. In such case, there were no more phosphate groups to coordinate with the uranyl ion, meaning that the adsorption balance was reached. Moreover, the equilibrium adsorption capacity of Fe_3_O_4_/P (GMA-MBA)-PO_4_ was much higher than that of Fe_3_O_4_/P (GMA-MBA), due to the fact that Fe_3_O_4_/P (GMA-MBA)-PO_4_ had a high concentration of phosphonic acid groups.

The uranium adsorption performance of Fe_3_O_4_/P (GMA-MBA) and Fe_3_O_4_/P (GMA-MBA)-PO_4_ at different temperatures is shown in Figure 4D. With the increase in contact temperature, the uranium adsorption capacity of the two adsorbents to uranium gradually increased, indicating that the uranium adsorption process was endothermic, and the increase in temperature was beneficial to the adsorption. To further investigate the effect of temperature on uranium adsorption performance, the three parameters including enthalpy changes ΔH° (KJ mol^−^^1^), ΔS° (J mol^−^^1^ K^−^^1^) and Gibbs free energy changes ΔG° (KJ mol^−^^1^) were studied. ΔH° and ΔS° were calculated using the thermodynamic formulas Equations (S1) and (S2). The ΔH° and ΔS° were calculated from the linear plots of lnK_d_ and 1/T (Figures S7 and S8). The ΔG° of Fe_3_O_4_/P (GMA-MBA) and Fe_3_O_4_/P (GMA-MBA)-PO_4_ were calculated, respectively, by using the Van’t Hoff equation. According to Table S3, since ΔH° was positive, the adsorption of Fe_3_O_4_/P (GMA-MBA)-PO_4_ to uranium was endothermic, suggesting the increase in the temperature was beneficial to the adsorption. The value of ΔS° was positive, indicating that the surface confusion and randomness of Fe_3_O_4_/P (GMA-MBA)-PO_4_ increased during the adsorption process. The value of ΔG° was negative, indicating that the uranium adsorption process was spontaneous.

Table S2 lists the sorption isotherm parameters of the Fe_3_O_4_/P (GMA-MBA) and Fe_3_O_4_/P (GMA-MBA)-PO_4_ calculated from the Langmuir and Freundlich models [41,42]. As shown in Figures S5 and S6 and Figures S5 and S6, the correlation coefficients R^2^ of Fe_3_O_4_/P (GMA-MBA) and Fe_3_O_4_/P (GMA-MBA)-PO_4_ were 0.995 and 0.9982 according to the Langmuir Model, which was much higher than that of Freundlich Model. The maximum adsorption capacity of Fe_3_O_4_/P (GMA-MBA)-PO_4_ for U (VI) (333.33 mg g^−^^1^) calculated by the Langmuir adsorption equation was closer to the experimental data (303.59 mg g^−^^1^). Compared with the Freundlich model, the Langmuir model was more compatible with the experimental data. Thus, it could be argued that the adsorption of Fe_3_O_4_/P (GMA-MBA)-PO_4_ for uranium was dominated by monolayer adsorption.

### 2.4. Structural Stability Analysis

**Figure 5:**
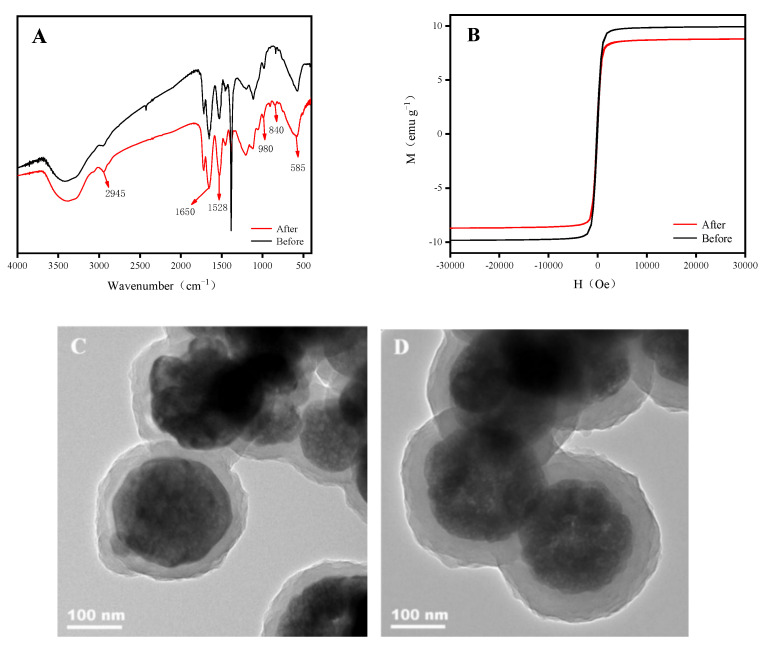
Fe_3_O_4_/P(GMA-MBA)-PO_4_ was soaked in a HNO_3_ medium with pH 4.5 for 24 h before and after: (**A**) Infrared spectra; (**B**) VSM spectrum; (**C**) TEM images taken before immersion; (**D**) TEM images after immersion.

To investigate the acid resistance of Fe_3_O_4_/P (GMA-MBA)-PO_4_, the adsorbent was soaked at a pH of 4.5 of HNO_3_ for 24 h, and then separated by an external magnet and freeze-dried. As shown in Figure 5, the soaked magnetic absorbent was characterized by FTIR, VSM and TEM. Figure 5A confirms that its structure did not significantly change after immersion and the surface functional group remained unchanged. The VSM test results are shown in Figure 5B, where the adsorbent magnetic response decreased slightly, but it still showed superparamagnetic character. The TEM images of the adsorbent before and after soaked are shown in Figure 5C,D. From Figure 5C,D, the material remained intact with a spherical structure after 24 h of soaking with no significant changes. The above results show that Fe_3_O_4_/P (GMA-MBA)-PO_4_ had a good structural stability at a pH of 4.5 of HNO_3_ for 24 h. 

### 2.5. Comparison of the U(VI) Sorption Capacity of Fe_3_O_4_/P (GMA-MBA)-PO_4_ with That of Other Adsorbents in Aqueous Solutions

As shown in Table 1, the Fe_3_O_4_/P (GMA-MBA)-PO_4_ of adsorption properties were compared with other adsorbents. From Table 1, the q_max_ of Fe_3_O_4_/P (GMA-MBA)-PO_4_ was up to 333.33 mg g^−1^, which was better than that of the other magnetic adsorbents listed in Table. For example, unmodified Fe_3_O_4_ had a uranium adsorption capacity of less than 50 mg g^−1^ at pH 7.0. After modifying the Fe_3_O_4_ (such as Fe_3_O_4_/GO, Fe_3_O_4_@C-KO, Fe_3_O_4_@TiO_2_, MCFN, et al.) uranium adsorption capacity increased, but its saturation adsorption capacity was still lower than that of Fe_3_O_4_/P (GMA-MBA)-PO_4_. Most magnetic adsorbents show a good adsorption capacity at pH 5.5–6.0, but the adsorption capacity decreased with the increase in acidity. Thus, Fe_3_O_4_/P (GMA-MBA)-PO_4_ could be regarded as a promising candidate for the separation and preconcentration of uranium.

### 2.6. Analysis of the Interactions between Uranium and Fe_3_O_4_/P(GMA-MBA)-PO_4_

**Figure 6:**
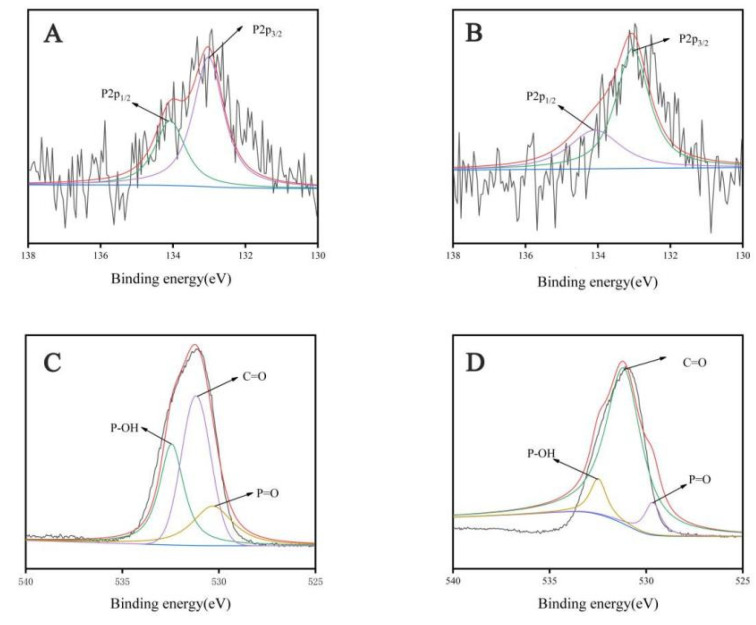
(**A**) P 2p map of Fe_3_O_4_/P (GMA-MBA)-PO_4_; (**B**) P 2p map of Fe_3_O_4_/P (GMA-MBA)-PO_4_-U; (**C**) O 1s map of Fe_3_O_4_/P (GMA-MBA)-PO_4_; (**D**) O 1s map of Fe_3_O_4_/P (GMA-MBA)-PO_4_-U.

To study the interaction between magnetic adsorbent and uranium ions, XPS analysis of Fe_3_O_4_/P (GMA-MBA)-PO_4_ before and after uranium adsorption is shown in Figure 6A–D. As shown in Figure 6A, the two peaks located at 133.9 eV and 133.0 eV were the characteristic peaks of P 2p1/2 and P 2p3/2. Compared with Figure 6A, the binding energy strength and position of Fe_3_O_4_/P (GMA-MBA)-PO_4_ of P 2p1/2 changed significantly (Figure 6B), indicating a new complexation between uranyl with P. As shown in Figure 6C, the O 1s spectra could be decomposed into three peaks, and the characteristic peaks at 530.3 eV, 531.2 eV and 532.5 eV belonged to P=O, C=O, and P-OH. After adsorption of Fe_3_O_4_/P (GMA-MBA)-PO_4_ for uranium, the position of the binding energy and strength of O 1s of P=O and P-OH bond changed significantly, while the position of O 1s of C=O bond remained almost unchanged (Figure 6D). Thus, it could be concluded that the adsorption process mainly depended on the interaction between the phosphonic acid group with uranium.

## 3. Materials and Methods

### 3.1. Materials

Bisacrylamide (MBA), O-phospethanolamine, γ-(Methacryloxypropyl) trimethoxy silane (KH570), glycidyl methacrylate (GMA) and azobisisobutyronitrile (AIBN) were bought from Shanghai Aladdin Biochemical Technology Co., Ltd. (Shanghai, China) Acetonitrile, nitrate acid (HNO_3_), sodium hyfroxide (NaOH) and FeCl_3_·6H_2_O were supplied from Xilong Chemical Co., Ltd. (Shantou, China) O-phospethanolamine was purchased from Shanghai Perlingway Chemical Technology Co., Ltd. (Shanghai, China) UO_2_ (NO_3_)_2_·6H_2_O (ACS grade) was purchased from Shanghai Reagents (Shanghai, China). All other chemicals used in the experiments were of analytical grade. Deionized water used for all experiments was supplied from a Milli-Q (Milli-pore Corporation, Burlington, MA, USA) water purification system.

### 3.2. Preparation of Fe_3_O_4_

Fe_3_O_4_ microspheres were prepared by the solvothermal method. The synthetic procedure was as follows: 2.5 g of FeCl_3_·6H_2_O was dissolved in 80 g of ethylene glycol (EG), and then 7.2 g of sodium acetate (NaAc) and 2.0 g of polyethylene glycol acid (PEG) were added to the solution. The mixture was stirred at 50 °C for 1 h and transferred to a 100 mL autoclave lined with polytetrafluoroethylene. The autoclave was heated to 200 °C for 6 h, and then left to cool naturally to room temperature. The Fe_3_O_4_ microspheres were washed with ethanol several times to remove the impurities under ultrasonic condition. Finally, Fe_3_O_4_ microspheres were kept at room temperature.

### 3.3. Preparation of Activated Fe_3_O_4_

In order to improve the coating rate of Fe_3_O_4_ in the polymer shell, the Fe_3_O_4_ hollow pellets were activated by γ-(Methacryloxypropyl) trimethoxy silane (KH570) [55]. The synthetic procedure was as follows: 0.3 g of Fe_3_O_4_, 128.0 mL of anhydrous ethanol, 4.0 mL γ-(Methacryloxypropyl) trimethoxy silane (KH570), 4.0 mL NH_3_·H_2_O and 36.0 mL of distilled water were added to a three-neck flask with a capacity of 250 mL. The mixture was stirred for 12 h at 40 °C. After the reaction, the product was washed several times to remove impurities. Finally, activated Fe_3_O_4_ microspheres by KH570 were kept at room temperature.

### 3.4. Preparation of Fe_3_O_4_/P (GMA-MBA)

Fe_3_O_4_/P(GMA-MBA) was prepared by the distillation and precipitation method. In a typical run, 0.10 g of surface-activated Fe_3_O_4_ microsphere and 80.0 mL acetonitrile were moved into 250 mL three-neck round bottom flask under ultrasonic oscillations. Then, 0.08 g of AIBN, 0.30 g of GMA and 0.30 g of MBA were added to the mixture. The mixture was heated to 90 °C for the reaction for 2 h. At the end of the reaction, the obtained magnetic microspheres were separated by an external magnetic field, washed several times and dried by lyophilization.

### 3.5. Preparation of Fe_3_O_4_/P (GMA-MBA)-PO_4_

In a typical run, Fe_3_O_4_/P (GMA-MBA) microspheres (0.1 g), O-phospethanolamine (1 g), DMF (50 mL) and H_2_O (50 mL) were added to a round-bottom flask. The mixture was stirred at 80 °C for 8 h. Then, the obtained Fe_3_O_4_/P (GMA-MBA)-PO_4_ was separated by an external magnetic field, washed several times and dried by lyophilization. The synthesis process is described in Scheme 1.

### 3.6. Characterization

The XRD pattern was measured with a D8 ADVANCE Da Vinci. FT-IR spectroscopy analysis were acquired on a Nicolet 6700 (Thermo Fisher, Waltham, MA, USA). Thermogravimetric analysis (TGA) was measured with an STA 449 F3 (Nichi, Bavaria, Germany). TEM images were performed on a TALOS F200X (Thermo Fisher, Waltham, MA, USA). X-ray photoelectron spectra (XPS) were acquired on an AXIS UItraDLD (Shimadzu, Kyoto, Japan). Magnetic properties (VSM) were measured on a MPMS3 (Quantum Design, San Diego, CA, USA).

### 3.7. Adsorption Tests

The adsorption experiments were carried out using the batch method to measure the adsorption property of uranium of the magnetic adsorbent. In a typical run, 10 mg of Fe_3_O_4_/P (GMA-MBA)-PO_4_ was placed into 25 mL of uranium solution at different pH values that could be adjusted by adding negligible volumes of diluted HNO_3_ or NaOH. After stirring for an appropriate time at a given temperature, the Fe_3_O_4_/P (GMA-MBA)-PO_4_ was collected by magnetic separation. The concentrations of UO_2_^2+^ in the aqueous solution, before and after adsorption, were determined by UV-vis spectrophotometer with arsenazo (III) as the complex agent. 

The adsorption capacity q_e_ (mg g^−1^) and the distribution coefficient, K_d_ (mL g^−1^), were calculated by Equations (1) and (2):

(1) $$q_{e}=\frac{\left(C_{0}-C_{e}\right)\times V}{M}$$

(2) $$K_{d}=\frac{C_{0}-C_{e}}{C_{0}}\times 100\%$$

where C_0_ and C_e_ are the initial concentration and equilibrium concentration of metal cations (mg L^−1^), K_d_ is the distribution coefficient (mL g^−1^), V is the volume of testing solution (L) and M is the amount of adsorbent (g).

### 3.8. Stability Tests

To investigate the structural stability of the magnetic adsorbent in an acidic solution, the prepared adsorbent was immersed at a pH of 4.5 of HNO_3_ for 24 h, and then was magnetically separated and freeze-dried with an external magnet. The adsorbent was subsequently characterized using TEM, FTIR, XRD and VSM to determine whether the structure had changed.

## 4. Conclusions

In this work, a novel phosphonic acid-functionalized core–shell magnetic polymer microsphere adsorbent Fe_3_O_4_/P (GMA-MBA)-PO_4_ was prepared by functionalized Fe_3_O_4_/P (GMA-MBA) with O-phosphoethanolamine. The physical and chemical properties and microstructure of the material were characterized by TEM, FT-IR, TG, XRD, XP and, VSM. The effect of pH, time, concentration and temperature on the adsorption behavior of the adsorbent was studied. The results showed that the adsorbent had a good adsorption performance for uranium, and its theoretical saturated adsorption capacity was up to 333.3 mg g^−1^. The study of adsorption kinetics and thermodynamics showed that the adsorption process was a fast, spontaneous and endothermic process, which accorded with the pseudo-second-order model and the Langmuir model. In addition, the adsorbent also had a good acid resistance and superparamagnetic character, which could be quickly separated by an external magnet. The XPS analysis showed that the adsorption process was mainly dependent on the interaction between the phosphonic acid group with uranium. Based on these, Fe_3_O_4_/P(GMA-MBA)-PO_4_ could be a promising candidate for the separation and preconcentration of uranium in actual water treatment.

## Figures and Tables

**Table 1:** Comparison of uranium adsorption performance between Fe_3_O_4_/P(GMA-MBA)-PO_4_ and other adsorbents.

Absorbent	q_max_ (mg g^−1^)	pH	Ref.
Fe-DETA	236.0	6.0	[43]
Amidoxime-modified Fe_3_O_4_@SiO_2_	105.0	5.0	[44]
Fe_3_O_4_/GO	69.5	5.5	[45]
Fe_3_O_4_@C-KO	38.7	6.0	[46]
Quercetin-modified Fe_3_O_4_ nanoparticles	12.3	3.7	[47]
Fe_3_O_4_@TiO_2_	118.8	6.0	[48]
Fe_3_O_4_@PAM	220.9	5.0	[49]
Fe_3_O_4_@SiO_2_	52.0	6.0	[50]
Phosphoramide-functionalized Fe_3_O_4_	95.2	6.0	[51]
Fe_3_O_4_	<50	7.0	[52]
MCFN	91.1	5.5	[53]
MNHA	310.0	5.0	[54]
Fe_3_O_4_/P(GMA-MBA)-PO_4_	333.3	4.5	This work

## Data Availability

Not applicable.

## Supplementary Materials

The supporting information can be downloaded at: https://www.mdpi.com/article/10.3390/ijms232416227/s1.

## References

[1] Yuan D., Zhang S., Tan J., Dai Y., Wang Y., He Y., Liu Y., Zhao X., Zhang M., Zhang Q. (2020). Highly efficacious entrapment of Th (IV) and U (VI) from rare earth elements in concentrated nitric acid solution using a phosphonic acid functionalized porous organic polymer adsorbent. Sep. Purif. Technol..

[2] Zhang Q., Zhang S., Zhao J., Wei P., Wang C., Liu P., Zhao X., Zeng K., Wu F., Liu Z. (2021). Unexpected ultrafast and highly efficient removal of uranium from aqueous solutions by a phosphonic acid and amine functionalized polymer adsorbent. New J. Chem..

[3] Zhang Q., Zeng K., Wang C., Wei P., Zhao X., Wu F., Liu Z. (2022). An imidazole functionalized porous organic polymer for the highly efficient extraction of uranium from aqueous solutions. New J. Chem..

[4] Mori T., Takao K., Sasaki K., Suzuki T., Arai T., Ikeda Y. (2015). Homogeneous liquid–liquid extraction of U (VI) from HNO3 aqueous solution to betainium bis(trifluoromethylsulfonyl)imide ionic liquid and recoveryof extracted U (VI). Sep. Purif. Technol..

[5] Zhang S., Zhao X., Li B., Bai C., Li Y., Wang L., Wen R., Zhang M., Ma L., Li S. (2016). “Stereoscopic” 2D super-microporous phosphazene-based covalent organic framework: Design, synthesis and selective sorption towards uranium at high acidic condition. J. Hazard. Mater..

[6] Venkatesan K.A., Shyamala K.V., Antony M.P., Srinivasan T.G., Rao P.R.V. (2008). Batch and dynamic extraction of uranium (VI) from nitric acid medium by commercial phosphinic acid resin, Tulsion CH-96. J. Radioanal. Nucl. Chem. Artic..

[7] Zhao Z., Wang X. (2021). Application of AnMBR Ion Exchange Technology in Water Treatment. IOP Conf. Series Earth Environ. Sci..

[8] Shimojo K. (2018). Solvent Extraction in Analytical Separation Techniques. Anal. Sci..

[9] Osmanlioglu A.E. (2018). Decontamination of radioactive wastewater by two-staged chemical precipitation. Nucl. Eng. Technol..

[10] Hao S., Jia Z., Wen J., Li S., Peng W., Huang R., Xu X. (2020). Progress in adsorptive membranes for separation—A review. Sep. Purif. Technol..

[11] Li H., Qing Q., Zheng L., Xie L., Gan Z., Huang L., Liu S., Wang Z., Lu Y., Chen J. (2022). Carbon dots and carbon nitride composite for photocatalytic removal of uranium under air atmosphere. Chin. Chem. Lett..

[12] Liu H., Mao Y. (2021). Graphene Oxide-based Nanomaterials for Uranium Adsorptive Uptake. ES Mater. Manuf..

[13] Gaillard C., Boltoeva M., Billard I., Georg S., Mazan V., Ouadi A. (2016). Ionic liquid-based uranium (VI) extraction with malonamide extractant: Cation exchange vs. neutral extraction. RSC Adv..

[14] Yuan Y., Zhao S., Wen J., Wang D., Guo X., Xu L., Wang X., Wang N. (2019). Rational Design of Porous Nanofiber Adsorbent by Blow-Spinning with Ultrahigh Uranium Recovery Capacity from Seawater. Adv. Funct. Mater..

[15] Luo W., Xiao G., Tian F., Richardson J.J., Wang Y., Zhou J., Guo J., Liao X., Shi B. (2019). Engineering robust metal–phenolic network membranes for uranium extraction from seawater. Energy Environ. Sci..

[16] Zhang S., Yuan D., Zhao J., Ren G., Zhao X., Liu Y., Wang Y., He Y., Ma M., Zhang Q. (2021). Highly efficient extraction of uranium from strong HNO_3_ media achieved on phosphine oxide functionalized superparamagnetic composite polymer microspheres. J. Mater. Chem. A.

[17] Zhang S., Yuan D., Zhang Q., Wang Y., Liu Y., Zhao J., Chen B. (2020). Highly efficient removal of uranium from highly acidic media achieved using a phosphine oxide and amino functionalized superparamagnetic composite polymer adsorbent. J. Mater. Chem. A.

[18] Wang X., Zhang J., Xu Z., Rao C., Pi L., Fu Y., Dong Y., Shen C., Yao L., Xiong C. (2021). Synthesis and application of recyclable core-shell structure microspheres MCTS-g-AT in detection of Hg (II) in aquatic products. J. Chin. Chem. Soc..

[19] Zhang Y., Du B., Wu Y., Liu Z., Wang J., Xu J., Tong Z., Mu X., Liu B. (2022). Fe_3_O_4_@PDA@PEI Core-Shell Microspheres as a Novel Magnetic Sorbent for the Rapid and Broad-Spectrum Separation of Bacteria in Liquid Phase. Materials.

[20] Zuo B., Li W., Wu X., Wang S., Deng Q., Huang M. (2020). Recent Advances in the Synthesis, Surface Modifications and Applications of Core-Shell Magnetic Mesoporous Silica Nanospheres. Chem. Asian J..

[21] Shi Z., Xu C., Lu P., Fan L., Liu Y., Wang Y., Liu L., Li L. (2018). Preparation and the adsorption ability of thiolated magnetic core-shell Fe_3_O_4_@SiO_2_@C-SH for removing Hg^2+^ in water solution. Mater. Lett..

[22] Chen G., Zhang G., Yang F. (2020). The elaboration of multifunctional hollow core-shell Fe_3_O_4_@PDA@TiO_2_ architecture with dual magnetic and photo-responsive performance. New J. Chem..

[23] Xu M., Han X., Wang T., Li S., Hua D. (2018). Conjugated microporous polymers bearing phosphonate ligands as an efficient sorbent for potential uranium extraction from high-level liquid wastes. J. Mater. Chem. A.

[24] Xu M.Y., Hai X.L., Hua D.B. (2017). Polyoxime-functionalized magnetic nanoparticles for uranium adsorption with high selectivity over vanadium. J. Mater. Chem. A.

[25] Calì E., Qi J., Preedy O., Chen S., Boldrin D., Branford W.R., Vandeperre L., Ryan M.P. (2018). Functionalised magnetic nanoparticles for uranium adsorption with ultra-high capacity and selectivity. J. Mater. Chem. A.

[26] Husnain S.M., Um W.Y., Chang W.L., Chang Y.S. (2018). Magnetite-based adsorbents for sequestration of radionuclides: A review. RSC Adv..

[27] Dai S., Wang N., Qi C., Wang X., Ma Y., Yang L., Liu X., Huang Q., Nie C., Hu B. (2019). Preparation of core-shell structure Fe3O4@C@MnO2 nanoparticles for efficient elimination of U (VI) and EU (III) ions. Sci. Total Environ..

[28] Song X.M., Tan L.C., Ma H.Y., Guo Y., Zhu L., Yi X.Q., Gao J.Y., Yang R.J., Dong Q. (2017). Facile preparation of S-doped magnetite hollow spheres for highly efficient sorption of uranium (VI). Dalton. Trans..

[29] Yuan D., Xiong X., Chen L., Lv Y., Wang Y., Yuan L., Liao S., Zhang Q. (2016). Removal of uranium from aqueous solution by phosphate functionalized superparamagnetic polymer microspheres Fe_3_O_4_/P(GMA-AA-MMA). J. Radioanal. Nucl. Chem..

[30] Yuan D., Chen L., Xiong X., Zhang Q., Liao S., Yuan L., Wang Y. (2016). Synthesis of PAMAM dendron functionalized superparamagnetic polymer microspheres for highly efficient sorption of uranium (VI). J. Radioanal. Nucl. Chem..

[31] Nogami M., Sugiyama Y., Ikeda Y. (2010). Adsorptivity of silica-supported adsorbents impregnated with polyphosphine polyoxides to U(VI) and some other metal ions in nitric acid media. J. Radioanal. Nucl. Chem. Artic..

[32] Broda E., Gładysz-Płaska A., Skwarek E., Payentko V.V. (2022). Structural properties and adsorption of uranyl ions on the nanocomposite hydroxyapatite/white clay. Appl. Nanosci..

[33] Biedrzycka A., Skwarek E., Osypiuk D., Cristóvao B. (2022). Synthesis of Hydroxyapatite/Iron Oxide Composite and Comparison of Selected Structural, Surface, and Electrochemical Properties. Materials.

[34] Biedrzycka A., Skwarek E., Hanna U.M. (2021). Hydroxyapatite with magnetic core: Synthesis methods, properties, adsorption and medical applications. Adv. Colloid Interface Sci..

[35] Li J., Guo Z., Zhang S., Wang X. (2011). Enrich and seal radionuclides in magnetic agarose microspheres. Chem. Eng. J..

[36] Yuan D., Zhang S., Xiang Z., He Y., Wang Y., Liu Y., Zhao X., Zhou X., Zhang Q. (2019). Highly Efficient Removal of Thorium in Strong HNO3 Media Using a Novel Polymer Adsorbent Bearing a Phosphonic Acid Ligand: A Combined Experimental and Density Functional Theory Study. ACS Appl. Mater. Interfaces.

[37] Jonsson M., Nyström D., Nordin O., Malmström E. (2009). Surface modification of thermally expandable microspheres by grafting poly(glycidyl methacrylate) using ARGET ATRP. Eur. Polym. J..

[38] Liu X., Sun J., Xu X., Alsaedi A., Hayat T., Li J. (2019). Adsorption and desorption of U(VI) on different-size graphene oxide. Chem. Eng. J..

[39] Zhang N., Yuan L.Y., Guo W.L., Luo S.Z., Chai Z.F., Shi W.Q. (2017). Extending the Use of Highly Porous and Functionalized MOFs to Th (IV) Capture. ACS Appl. Mater. Interfaces.

[40] Han X., Wang Y., Cao X., Dai Y., Liu Y., Dong Z., Zhang Z., Liu Y. (2019). Adsorptive performance of ship-type nano-cage polyoxometalates for U (VI) in aqueous solution. Appl. Surf. Sci..

[41] Wang D., Xu Y., Xiao D., Qiao Q., Yin P., Yang Z., Li J., Winchester W., Wang Z., Hayat T. (2019). Ultra-thin iron phosphate nanosheets for high efficient U(VI) adsorption. J. Hazard. Mater..

[42] Liu Y., Ouyang Y., Huang D., Jiang C., Liu X., Wang Y., Dai Y., Yuan D., Chew J.W. (2020). N, P and S co-doped carbon materials derived from polyphosphazene for enhanced selective U (VI) adsorption. Sci. Total Environ..

[43] Amesh P., Suneesh A.S., Selvan B.R., Venkatesan K.A., Chandra M. (2020). Magnetic assisted separation of uranium (VI) from aqueous phase using diethylenetriamine modified high capacity iron oxide adsorbent. J. Environ. Chem. Eng..

[44] Zhao Y., Li J., Zhao L., Zhang S., Huang Y., Wu X., Wang X. (2014). Synthesis of amidoxime-functionalized Fe3O4@SiO2 core–shell magnetic microspheres for highly efficient sorption of U(VI). Chem. Eng. J..

[45] Zong P., Wang S., Zhao Y., Wang H., Pan H., He C. (2013). Synthesis and application of magnetic graphene/iron oxides composite for the removal of U(VI) from aqueous solutions. Chem. Eng. J..

[46] Liu Q., Li W., Zhao W., Tan L., Jing X., Liu J., Song D., Zhang H., Li R., Liu L. (2016). Synthesis of ketoxime-functionalized Fe3O4@C core–shell magnetic microspheres for enhanced uranium(vi) removal. RSC Adv..

[47] Sadeghi S., Azhdari H., Arabi H., Moghaddam A.Z. (2012). Surface modified magnetic Fe3O4 nanoparticles as a selective sorbent for solid phase extraction of uranyl ions from water samples. J. Hazard. Mater..

[48] Tan L., Zhang X., Liu Q., Jing X., Liu J., Song D., Hu S., Liu L., Wang J. (2015). Synthesis of Fe3O4@TiO2 core–shell magnetic composites for highly efficient sorption of uranium (VI). Colloids Surfaces A Physicochem. Eng. Asp..

[49] Song W., Liu M., Hu R., Tan X., Li J. (2014). Water-soluble polyacrylamide coated-Fe3O4 magnetic composites for high-efficient enrichment of U(VI) from radioactive wastewater. Chem. Eng. J..

[50] Fan F.L., Qin Z., Bai J., Rong W.D., Fan F.Y., Tian W., Wu X.L., Wang Y., Zhao L. (2012). Rapid removal of uranium from aqueous solutions using magnetic Fe3O4@ SiO2 composite particles. J. Environ. Radioact..

[51] Singhal P., Vats B.G., Yadav A., Pulhani V. (2020). Efficient extraction of uranium from environmental samples using phosphoramide functionalized magnetic nanoparticles: Understanding adsorption and binding mechanisms. J. Hazard. Mater..

[52] Das D., Sureshkumar M., Koley S., Mithal N., Pillai C. (2010). Sorption of uranium on magnetite nanoparticles. J. Radioanal. Nucl. Chem..

[53] Li L., Huang F., Yuan Y., Hu J., Tang Q., Tang S. (2013). Preparation and sorption performance of magnetic 18-crown-6/Fe3O4 nanocomposite for uranium (VI) in solution. J. Radioanal. Nucl. Chem..

[54] El-Maghrabi H.H., Younes A.A., Salem A.R., Rabie K., El-Shereafy E.S. (2019). Magnetically modified hydroxyapatite nanoparticles for the removal of uranium (VI): Preparation, characterization and adsorption optimization. J. Hazard. Mater..

[55] Zhang Y., Yang Y., Ma W., Guo J., Lin Y., Wang C. (2013). Uniform Magnetic Core/Shell Microspheres Functionalized with Ni^2+^–Iminodiacetic Acid for One Step Purification and Immobilization of His-Tagged Enzymes. ACS Appl. Mater. Interfaces.

