# Supplementary material for: Highly Efficient Removal of Uranium from an Aqueous Solution by a Novel Phosphonic Acid-Functionalized Magnetic Microsphere Adsorbent
Source: Int J Mol Sci. 2022 Dec 19;23(24):16227. doi: 10.3390/ijms232416227 (PMC9787024; doi:10.3390/ijms232416227)
Supplement: Supplementary file 1 [file ijms-23-16227-s001.zip › ijms-1970397-supplementary.pdf]

Highly Efficient Removal of Uranium from an Aqueous Solution by a Novel Phosphonic Acid-Functionalized Magnetic Microsphere Adsorbent

Jizhou Zhao, Peng Lu, Tengteng He, Jing Huang, Shiao Zhang, Yan Liu, Yun Wang, Cheng Meng and Dingzhong Yuan *

Jiangxi Province Key Laboratory of Polymer Micro/Nano Manufacturing and Devices, East China University of Technology, Nanchang 330013, China

* Correspondence: 201060026@ecut.edu.cn

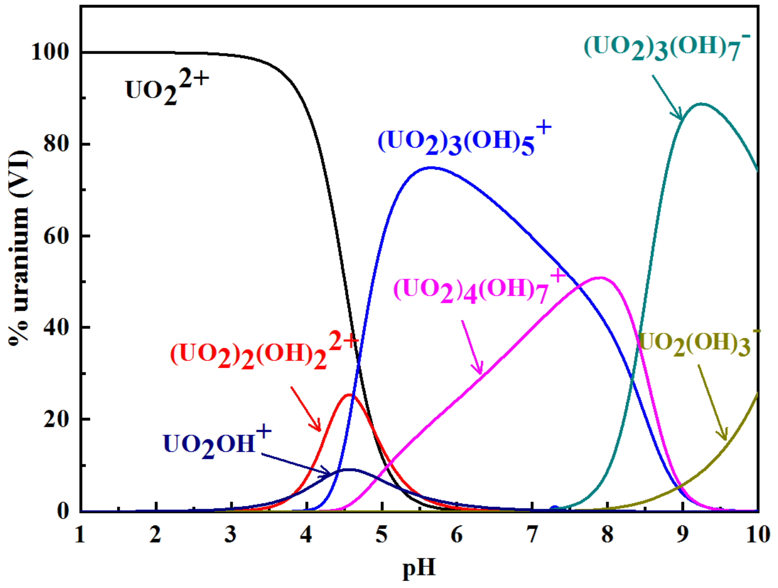

Figure S1. Distribution of uranium (VI) species in aqueous solution with a total concentration of 100 mg L^{-1} and pH values ranging from 1 to 10. Calculated by using a Medusa program.

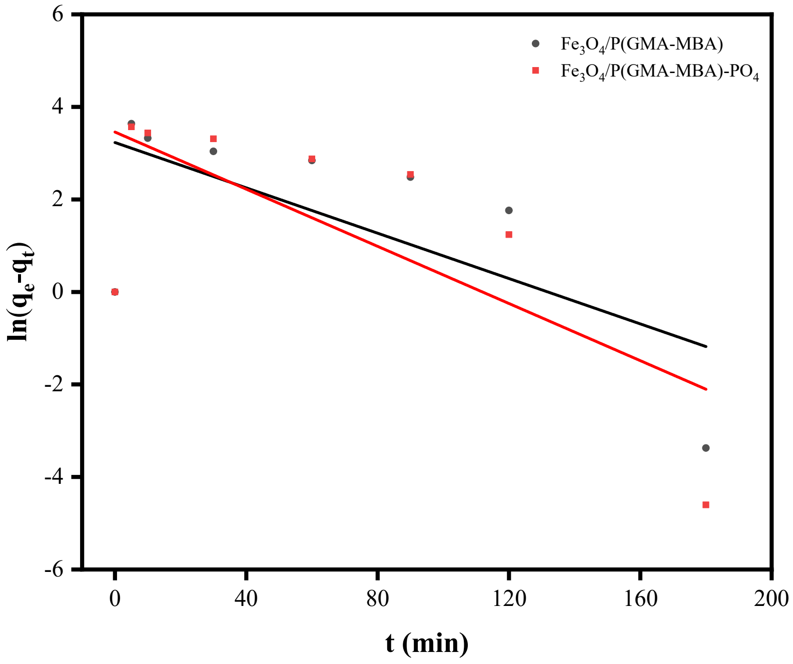

Figure S2. Pseudo-first-order model.

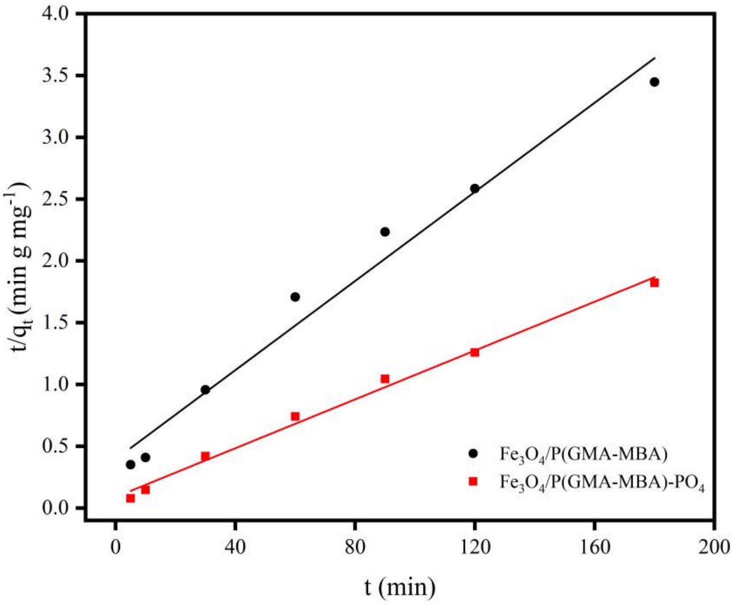

Figure S3. Pseudo-second-order model.

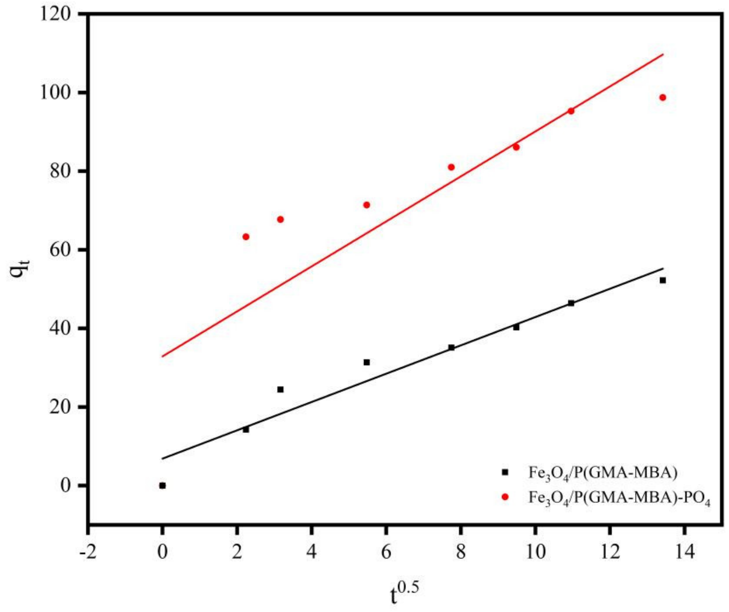

Figure S4. Intra-particle diffusion model.

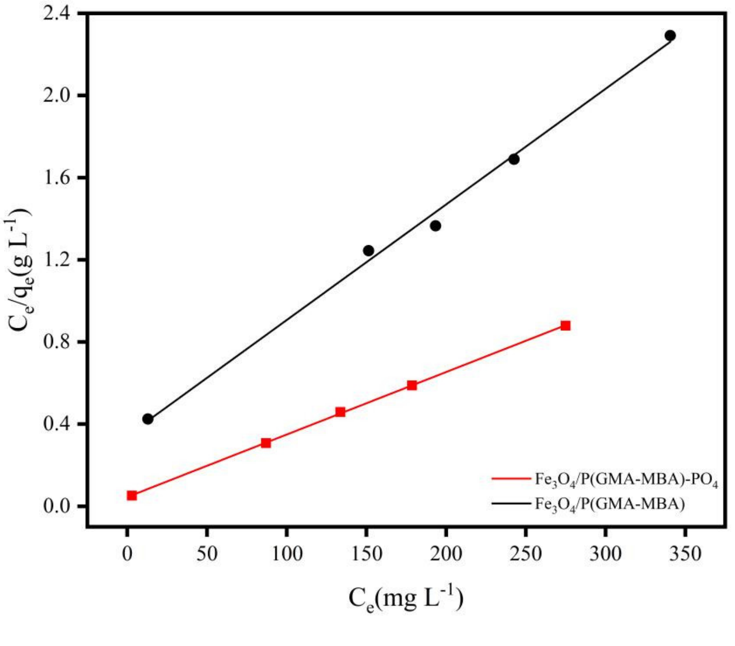

Figure S5. Langmuir modal.

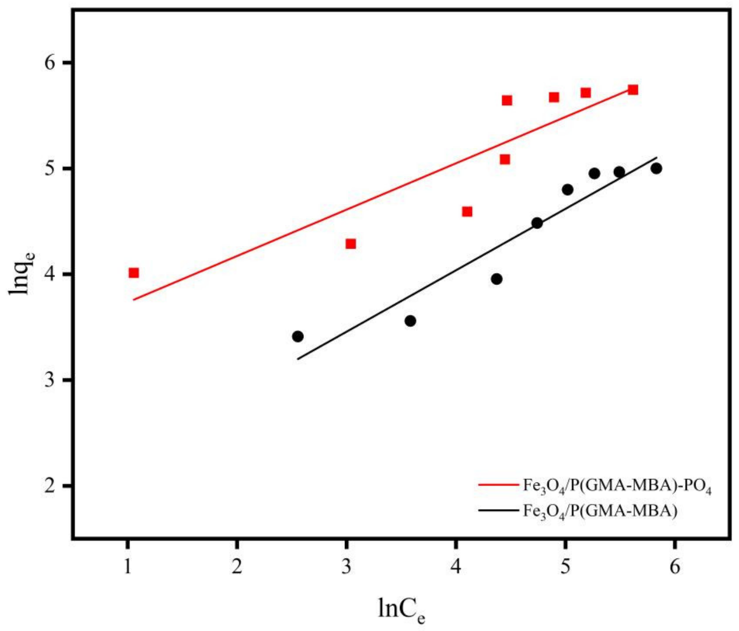

Figure S6. Freundlich modal.

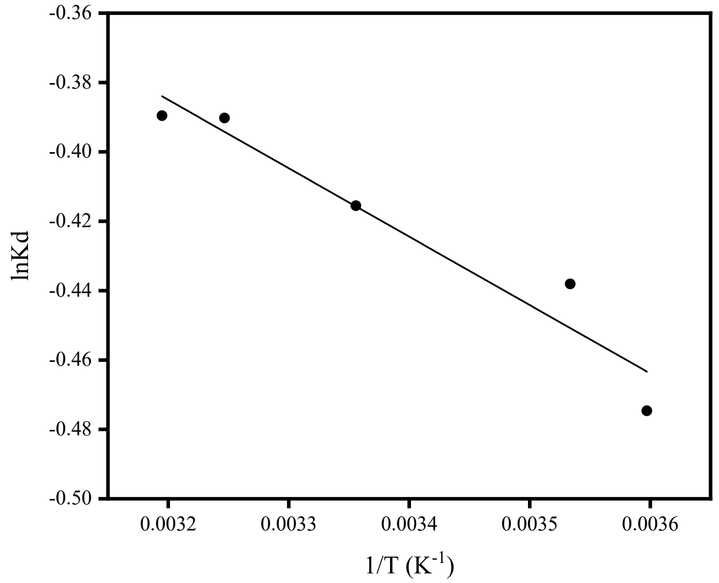

Figure S7. Thermodynamic image of U (VI) adsorbed by Fe_3O_4/P (GMA-MBA).

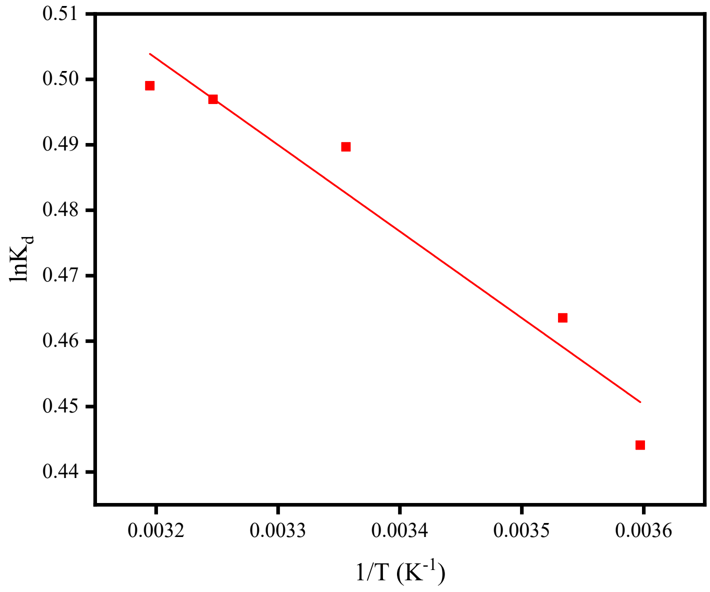

Figure S8. Thermodynamic image of U (VI) adsorbed by

Fe_3O_4/P(GMA-MBA)-PO_4

Table S1. Related kinetic parameters of uranium (VI) adsorption by Fe_3O_4/P(GMA-MBA) and Fe_3O_4/P(GMA-MBA)-PO_4.

Kinetic model	Parameter	Fe_3O_4/P(GMA-MBA)	Fe_3O_4/P (GMA-MBA)-PO_4
Pseudo-first-order	k_1 (1 sec^{-1})	0.0245	0.0309
	$q_{e.\ cal}$ (mg g^{-1})	20.28	31.70
	R^2	0.4478	0.5121
Pseudo-second-order	k_2 [g (mg^{-1} sec^{-1})]	0.0012	0.0011
	$q_{e.\ cal}$ (mg g^{-1})	52.91	101.21
	R^2	0.9729	0.9931
Intraparticle diffusion	K_{int} [mg (g^{-1} sec$^{-1/2}$)]	3.6017	5.7234
	c (mg g^{-1})	6.8808	32.894
	R^2	0.9430	0.7266

Table S2. Fe_3O_4/P(GMA-MBA) and Fe_3O_4/P(GMA-MBA)-PO_4 adsorption isotherm parameters for uranium.

Model	Parameter	Fe_3O_4/P(GMA-MBA)	Fe_3O_4/P (GMA-MBA)-PO_4
Langmuir	b (L mg $^{-1}$)	0.016	0.066
	q_{max} (mg g^{-1})	178.57	333.33
	R^2	0.995	0.9982
Freundlich	K_F (mg g^{-1})	5.77	27.042
	n_F	1.72	2.28
	R^2	0.9125	0.8051

Table S3. Thermodynamic parameters of Uranium adsorption by Fe_3O_4/P(GMA-MBA) and Fe_3O_4/P(GMA-MBA)-PO_4

**Absorbent**	**ΔH° (KJ mol⁻¹)**	**ΔS° (J mol⁻¹ K⁻¹)**	**ΔG° (KJ mol⁻¹)**				
			278K	283 K	298 K	308 K	313 K
Fe_3O_4/P(GMA-MBA)	1.640	2.048	-0.57	-0.58	-0.61	-0.63	-0.64
Fe_3O_4/P(GMA-MBA)-PO_4	4.514	7.702	-1.04	-1.12	-1.20	-1.28	-1.35

$$\ln K^{\circ} = \frac{\Delta S^{\circ}}{R} - \frac{\Delta H^{\circ}}{RT}$$ Equation (S1)

$$\Delta G^{\circ} = \Delta H^{\circ} - T\Delta S^{0}$$ Equation (S2)

where ΔH°, ΔS°, and ΔG° stands for the enthalpy (KJ mol $^{-1}$), entropy (J mol^{-1} K^{-1}) and Gibbs free energy (KJ mol $^{-1}$). K° is the sorption equilibrium constant (mL g^{-1}).
